# Stress, depression, and anxiety: psychological complaints across menopausal stages

**DOI:** 10.3389/fpsyt.2024.1323743

**Published:** 2024-02-22

**Authors:** Ming Jun Kuck, Eef Hogervorst

**Affiliations:** School of Sport, Exercise, and Health Sciences, Loughborough University, Loughborough, United Kingdom

**Keywords:** menopause, perceived stress, resilience, psychological complaints, early perimenopause, depression, anxiety, self efficacy

## Abstract

**Introduction:**

With the number of menopausal women projected estimated to reach 1.2 billion by 2030 worldwide, it is critically important to understand how menopause may affect women’s emotional well-being and how many women are affected by this. This study aimed to explore (i) the relationship between psychological complaints (depression, anxiety, poor memory) across different menopausal stages and (ii) investigate the correlation between resilience, self-efficacy, and perceived stress levels, with psychological complaints and whether this was associated with menopausal stage and/or age.

**Methods:**

287 respondents completed the Menopausal Quality of Life (MenQoL), Perceived Stress Scale (PSS-10), Brief Resilience Scale (BRS), and General Self-efficacy (GSE) scales. Parametric and non-parametric analysis were used to analyse how bothered women were by self-reported poor memory and feelings of depression and anxiety, alongside perceived stress, resilience, and self-efficacy between women in different menopausal stages using STRAW criteria. The association between protective factors (self-efficacy and resilience) and psychological complaints was analysed with partial correlation analysis controlling for menopausal stages and/or age.

**Results:**

A significant difference was found between the levels of perceived stress, and how bothered women were by feelings of depression and anxiety between early-perimenopausal and post-menopausal women. However, with the inclusion of age as a covariate, menopausal stage no longer predicted the level of self-reported stress and anxiety in menopausal women. There was also no difference between poor self-reported memory, or of self-efficacy or resilience between women in different menopausal stages. However, self-efficacy and resilience were associated with how bothered women were by feelings of depression and anxiety, and the experience of stress. Stress was the only variable to be associated with poor self-reported memory independent of age and/or menopausal status.

**Discussion:**

Early perimenopausal women experienced the highest level of stress and were more severely bothered by feelings of depression and anxiety, with the poorest overall self-reported psychosocial quality of life. Post-menopausal women, however, reported to have similar experiences as premenopausal women. Age explained the associations between menopausal stage, stress and anxiety, but not between depression and different menopausal stages. Resilience and self-efficacy were associated with psychological complaints independent of menopausal stage and age, suggesting that therapies focusing on increasing resilience and self-efficacy may be beneficial to help target these psychological complaints at any time.

## Introduction

Menopause is a significant milestone in life, marking the end of one’s reproductive years. Women experience natural menopause as their levels of oestrogen and progesterone change and ultimately decline as a part of biological ageing, typically between the ages of 45 and 55, with the final menstrual period occurring on average around the age of 51 (1). The menopausal transition consists of several stages: pre-menopause, early perimenopause, late perimenopause, and post-menopause (1). According to the Stages of Reproductive Aging Workshop +10 (STRAW) criteria (1): *“Premenopausal”* refers to having a regular menstrual cycle, with menstrual bleeding occurring in the past three months. *“Early perimenopause”* is marked by a more irregular menstrual cycle over the past year, but with women still experiencing menstrual bleeding within the last three months. *“Late perimenopause”* occurs when menstrual bleeding has not occurred in the last three months, but has happened in the previous 12 months. Lastly, *“Post-menopause”* is the absence of menstrual bleeding in the last 12 months, not caused by medication, pregnancy, or severe weight loss (1).

This inevitable life stage can be accompanied by various menopausal symptoms, such as hot flushes, night sweats, brain fog, and sleep disturbances, which patterns are unique to every woman (2–4). As reported by The Fawcett Society in 2022, over 77% of women from the UK undergoing the menopausal transition report at least one menopausal symptom (hot flushes being the most prevalent), and 44% experience three or more severe symptoms (2). Menopausal symptoms are estimated to lead to a global loss of $150 billion per year globally due to reduced work productivity, which is reported by 1 in 3 women during the menopausal transition (5). Those women who suffer from severe menopausal symptoms often have a heightened level of presenteeism and overall report increased difficulties with work, compared to those who do not experience these symptoms (6).

Research has shown that within the menopausal transition, the prevalence and severity of vasomotor (VMS such as hot flushes and night sweats) and sexual menopausal symptoms are closely tied to the stage of the menopausal transition (3, 7). An increasing occurrence and frequency of VMS symptoms with advancement during the menopausal stages is observed, where the peak is seen in the late perimenopause and postmenopausal years (3, 7, 8). This may result from fluctuations in hormone levels or occur as part of biological ageing, as age was shown to be associated with the severity of vasomotor and sexual symptoms in a recent study (9, 10). On the other hand, reduced oestradiol levels and elevated follicle-stimulating hormone (FSH), often used as markers of menopause, were shown to be associated with an increase in the prevalence and severity of vasomotor and sexual symptoms, independent of age (11–13). Menopausal complaints in the vasomotor and sexual domain reported by postmenopausal women are usually well treated with hormone replacement therapy (HRT) (14, 15). The literature to date has mainly focused on the difference in vasomotor and sexual symptoms between menopausal stages but increasing attention is given to psychological complaints associated with the menopausal transition.

Over half of British women reported that they also struggled with anxiety and depression during the menopausal transition (2). The relationship between these psychological symptoms and menopausal stages remains unclear. Some suggest these are perhaps more associated with general psychosocial factors presented in midlife, such as career- and family issues including marital discord, empty nest syndrome, difficult teenagers and care for older parents (16, 17). Unlike vasomotor and sexual symptoms, psychological symptoms may not increase with advancement in the menopausal stages, as higher reported rates of psychological complaints such as depression were observed in perimenopausal women compared to premenopausal and postmenopausal women (18, 19). It is unclear whether these psychological issues decrease after the perimenopausal stage and whether that is indicative of habituation to lower oestrogen levels (19).

In terms of clinically diagnosed disorders, which should significantly affect activities of daily life including work according to consensus based psychiatric criteria, some literature suggests that women with a history of major depressive disorder (MDD, diagnosed using DSM: Diagnostic and Statistical Manual of Mental Disorders) are at a higher risk of developing MDD during the menopausal transition (20). Research showing that perimenopausal women were more prone to depression than premenopausal women, even proposed the concept of ‘perimenopausal depression’ as a unique subtype of depressive disorders (19, 21). This underlines the importance of examining the experienced severity of psychological complaints during the menopausal transition and its impact on health, work and quality of life (11, 22, 23). In contrast Amore et al. (24) found a greater severity of depressive symptoms using the women’s health questionnaire (so not measuring clinical disorders) in the postmenopausal group compared to peri- and premenopausal women. However, more recent cross-sectional studies found no significant difference in the prevalence of depressed mood between women of different menopausal stages (25, 26).

Depression is often co-morbid with anxiety disorders and can present with memory complaints (27, 28). A survey in 2022 reported that 69% of British women described anxiety as being a ‘very’ or ‘somewhat difficult’ symptom during the menopausal transition (similarly assessed as in the MenQol) (2). Although the relationship between depression and menopause has been studied as outlined, research on anxiety is substantially neglected (29–31). A systematic review of anxiety during the menopausal transition suggested that the relationship between anxiety and menopause transition could not really be determined due to substandard and unstandardised measures of anxiety across various studies (29). Women are often not aware or informed about the possible detrimental effects of menopause on their emotional and mental health, especially during the early stages of transition (2). This study aims to help identify the stage during the menopausal transition when women are the most vulnerable, as understanding and acknowledgement of these issues may be beneficial to them.

Alongside psychological complaints (depression, anxiety, and memory issues), perceived (low) stress, resilience, and self-efficacy were also measured in this present study, as psychological complaints are closely tied to such potentially protective psychosocial factors (16, 17). Past research found that less-resilient individuals showed higher levels of psychopathological symptoms, such as those found in depression and anxiety disorders (32, 33). Süss and Ehlert (34) proposed resilience as a vital piece in one’s cognitive appraisal and emotional experience of the climacteric symptoms, which leads to successful or unsuccessful adjustment. In addition, recent studies found that an individual’s level of self-efficacy may affect their experience of menopausal symptoms and vice versa (35, 36). This again underlines the importance of measuring how bothered women going through menopausal transition are by these psychological complaints instead of solely measuring whether they experience these issues or have a clinical disorder and whether degree of bother may be off-set by protective factors such as resilience and self-efficacy.

Perceived stress is measured as previous research has established that this may worsen menopausal physical, vasomotor, sexual and psychological symptoms (36, 37). The relationship between low perceived stress and different menopausal stages remains ambiguous, with mixed results reported by studies (38, 39). Hence, this study also aims to find out the correlation between resilience, self-efficacy, and low perceived stress, with how severely bothered women were by poor memory and feelings of depression, and anxiety between different menopausal stages to identify potential treatments to improve women’s menopausal quality of life (40).

The main objectives of this study were to: (i) examine the relationship between psychological complaints (such as bother perceived by depression, anxiety, and poor memory) and protective factors (resilience, self-efficacy and low perceived stress) in different menopausal stages of women to identify the particular stage of menopausal transition where women may need the most support; and (ii) investigate the association between resilience, self-efficacy, perceived stress levels, *vs*. how bothered women were by feelings of anxiety, depression, and poor memory to identify possible interventions to optimize quality of life during menopausal transition.

## Materials and methods

### Participants

Middle-aged individuals between the ages of 40 and 60 years old were recruited through convenience sampling using social media and other platforms such as Women in Higher Education Network and other related web-based groups. Participants received a link to the online survey built using the Bristol Online Surveys platform. All data collected were anonymous, and no incentive was provided. Ethics approval was awarded by the Loughborough University Ethics Review Sub-Committee (Project ID: 13264). Prior to participating, participants reviewed an information sheet and provided their consent. They had the option to withdraw from the study at any point during the survey, and their data were not retained.

### Assessments

General socio-demographic information was gathered, including age, ethnicity, highest level of education, first language, marital status, number of children, and current employment status. This was followed by questions regarding their menopausal stages, type of menopause, and type and age at starting hormone therapy for menopause. Finally, four established questionnaires were administered in the following order:

#### Perceived stress scale

The PSS-10 (41) is a 5-point Likert scale which assesses one’s experience of stress-related feelings and thoughts within the last month. The PSS-10 consists of four positive items and six negative items. Women can grade each item from 0 (never) to 4 (very often). The sum is calculated after reversing the four positive items’ scores, with a higher score indicating higher perceived stress. Cronbach’s Alpha for the scale was α=.87 in the present study.

#### Brief resilience scale

The BRS (42) comprises 6 Likert items, with half of the items reversely coded to ensure high internal validity of the scale. The scale measures one’s subjective perception of their ability to bounce back or cope with stress. Participants rank each item from ‘strongly disagree’ to ‘strongly agree’. The resilience score is calculated by dividing the sum by the total number of questions answered, with a higher score indicating a higher level of resilience. The internal consistency of the scale in this study was good, with α=.82.

#### Menopause specific quality of life

Psychological complaints such as depression, anxiety, and memory issues were measured through the MenQoL (43). It consists of four domains with 29 items: vasomotor, psychosocial, physical, and sexual. The specific questions used to measure how bothered women were by poor memory and feelings of depression and anxiety are as followed: “Experiencing poor memory” “Feeling depressed, down or blue”, “Feeling anxious or nervous”. Participants selected ‘Yes’ or ‘No’, to indicate whether they had experienced the problem in the past month, followed by how bothered they were from 0 (not at all bothered) to 6 (extremely bothered). Participants received a conversion score of 1 when they did not experience the item and 0 if they did experience it. The conversion score ranged from 1 to 8, with ‘2’ meaning the participant experienced the item but was not at all bothered by it, and ‘8’ was where the participant was extremely bothered by the experience of the item. Each domain is scored separately, and the overall questionnaire score is the average of the domain means. A lower overall mean indicates a better quality of life. The Cronbach’s Alpha coefficient for each domain was vasomotor α=.77, psychosocial α=.83, physical α=.87, and sexual α=.69.

#### General self-efficacy scale

The GSE (44) consists of 10 items on a 4-point scale to assess one’s sense of perceived self-efficacy (control). It had a high internal consistency of α=.90 in this study. The GSE helps predict how well a person will cope with daily stressors and adapt after experiencing various stressful life events. A total score ranging from 10 to 40 is calculated by summing up the responses to all ten items, with no recoding required. A higher total score represents a higher level of perceived self-efficacy, and vice versa; a lower total score represents a lower level of self-efficacy.

### Data analysis

All statistical analyses were conducted using the Statistical Package for the Social Sciences (SPSS), version 28. The descriptive statistical analysis examined participants’ resilience, self-efficacy, perceived stress, and MenQoL sub-scales and questions per menopausal transition stage. Preliminary analyses were conducted, to ensure no assumptions were violated for the following tests. A partial correlation analysis was applied to measure the associations between variables using menopause and/or age as a covariate. The normality of data distribution was tested with the Kolmogorov-Smirnov tests. According to this outcome, non-parametric data were compared with Kruskal-Wallis tests, and ANOVA was used for parametric comparisons using the menopausal stage as an independent variable. Covariates such as age and other general socio-demographic information which were associated with menopausal stage were included in ANOVA or GLM. For all calculations, *P*<0.05 was considered statistically significant.

## Results

Out of 287 responses received, 14 birth-assigned males and one woman who failed to complete the survey were excluded. The remaining 272 women with a mean age of (*M*=51.26) were categorised based on the STRAW criteria: premenopausal (*N*=37), early perimenopausal (*N*=80), late perimenopausal (*N* =40), and postmenopausal (*N* =115). Twenty women who underwent surgical menopause were included and grouped with postmenopausal women due to the small sample size. Among these 20 women, 11 underwent bilateral oophorectomies at the average age range of menopause (50/51 years of age) during the testing period. Only four women experienced premature menopause and received hormone therapy. Analyses leading to combining both groups had not shown a difference between surgical and naturally postmenopausal women across the variables of interest (see **Supplementary Table 1**. for the results of the separate analysis between variables for natural and surgical menopausal women). The general characteristics of the participants are depicted in **Table 1**. The majority of the sample were White British and White others (90.4%), employed full-time or part-time (81.7%), in a relationship (75.7%), and current or past Hormone Replacement Therapy (HRT) was reported by 60.7%.

**Table 1:** Sociodemographic characteristics of the sample.

Sample characteristics	N (%)
Age (years)	
40-44	25 (9.3%)
45-49	67 (25%)
50-54	107 (39.9%)
55-60	69 (25.7%)
Race/Ethnicity	
White British/Other White Background	246 (90.4%)
Black/African/Caribbean/Black British	3 (1.1%)
Asian/Asian British	16 (5.9%)
Mixed/Multiple Ethnic groups	4 (1.5%)
Other	3 (1.1%)
Level of education	
Less than high school	10 (3.7%)
High school/College/A-Levels	86 (31.6%)
University degree	99 (36.4%)
Postgraduate degree	77 (28.3%)
Employment Status	
Employed full-time	153 (56.3%)
Employed part-time	69 (25.4%)
Retired	8 (2.9%)
Other	41 (15.1%)
Marital Status	
Married/In a relationship	206 (75.7%)
Divorced/Separated/Single	62 (22.8%)
Widowed	3 (1.1%)
Number of Children	
None	62 (22.8%)
1	44 (16.2%)
2-4	161 (59.2%)
More than 4	5 (1.8%)
HRT use	
None	107 (39.9%)
Past	22 (8.1%)
Current	143 (52.6%)
Menopausal Stage	
Premenopausal	37 (13.6%)
Early perimenopausal	80 (29.4%)
Late perimenopausal	40 (14.7%)
Postmenopausal	115 (42.3%)

**Figure 1:**
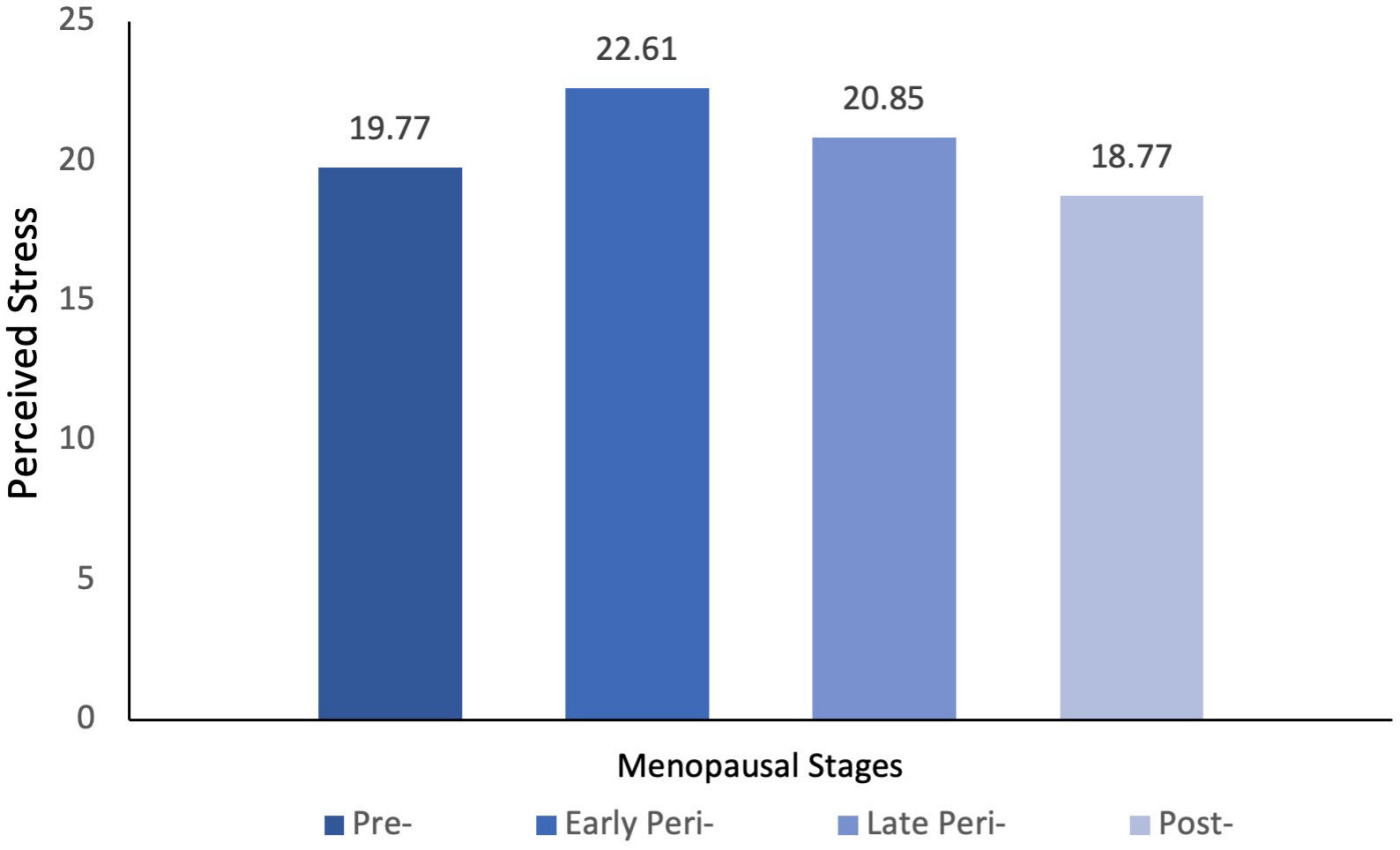
Differences in the level of perceived stress between menopausal groups.

A one-way ANOVA or general linear model (GLM) was performed on perceived stress as a dependent variable against the menopausal stages of women. As shown in **Table 2**, a statistically significant difference in the level of perceived stress was detected between menopausal stages. *Post hoc* comparison using the Tukey HSD test indicated that the mean score for perceived stress was only significantly different between early perimenopausal and postmenopausal women (see **Figure 1**). These results suggested that early perimenopausal women experienced a higher level of stress than postmenopausal women, whilst there was no significant difference in the level of perceived stress between the other menopausal stages (*p*>0.05). However, with the inclusion of age as a covariate in the GLM, the differences in perceived stress level between menopausal stages were no longer significant (*p*>0.05). Further Pearson correlation coefficients were computed to assess the linear relationship between age and perceived stress. A negative correlation between the two variables (r(268)=-.20, p<.001) was shown, with older women suffering less from stress. Inclusion of covariates such as education, marital status, number of children, employment status, and HRT use associated with menopausal stage did not change the model and did not contribute to the model independently.

**Table 2:** Descriptive statistics of participants’ resilience, perceived stress, self-efficacy, psychological complaints (feeling depressed, down or blue; feeling anxious or nervous, and experiencing poor memory) as measured by Menopausal Quality of Life Scale, and psychosocial domain of quality of life.

	Mean (*SD*)			
	Premenopausal	Early perimenopausal	Late perimenopausal	Postmenopausal
Resilience	3.10 (1.00)	3.12 (0.98)	3.28 (0.88)	3.28 (0.74)
Self-efficacy	29.20 (6.30)	29.57 (5.14)	28.60 (4.63)	29.81 (4.54)
Perceived Stress	19.77 (7.52)	22.61 (7.72) **	20.85 (7.15)	18.77 (7.00) **
Depression	2.97 (2.40) *	4.80 (2.61) */**	4.10 (2.65)	3.61 (2.46) **
Anxiety	4.43 (2.32)	5.23 (2.45) **	4.47 (2.73)	4.23 (2.62) **
Psychosocial	3.58 (1.76) *	4.67 (1.93) */**	4.28 (1.72)	3.73 (1.72) **
Poor memory	3.66 (2.45)	4.65 (2.64)	4.97 (2.61)	4.40 (2.42)

Dependent variables: Perceived stress scale (total score from 0-40), Menopausal Quality of Life Scales (score from 1-8).

* Significant difference between pre- vs early peri-

** Significant difference between early perimenopause and post-menopause only.

**Figure 2:**
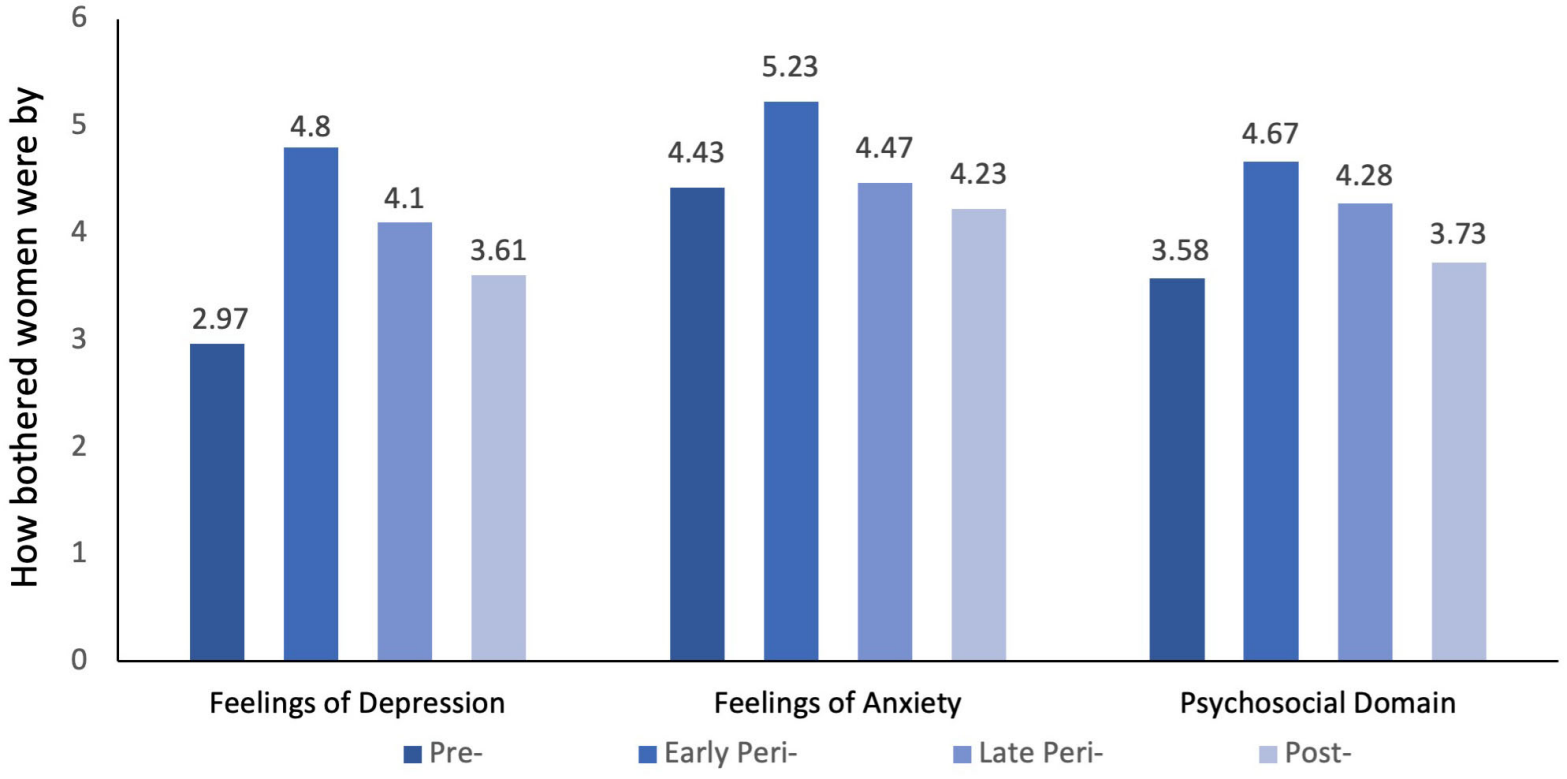
Menqol scores for how bothered women were by feelings of depression, anxiety, and the psychosocial domain between menopausal groups.

For variables that did not meet the assumption for parametric analysis, the non-parametric independent-samples Kruskal-Wallis Test was applied. A significant difference in the severity of how bothered women were by feelings of depression (H(3)=15.689, p=.001), anxiety (H(3)=8.076, p=.044), and the total psychosocial domain (which included poor memory) measured by the MenQoL scale (H(3)=15.315, p=.002) by menopausal stages was detected, as shown in **Figure 2**. Further *post hoc* pair-wise comparison tests indicated that the severity of how bothered women were by feelings of depression significantly differed between premenopausal women and early perimenopausal women (p<0.05) and postmenopausal and early perimenopausal women (p<0.05). Early perimenopausal women reported a higher prevalence and were more severely bothered by feelings of depression than premenopausal and postmenopausal women (p<0.05). Severity level of how bothered women were by feelings of anxiety was also significantly different between postmenopausal and early perimenopausal women (p=0.006). Women in the early perimenopausal stage were more likely to be bothered by the feelings of anxiety and nervousness than postmenopausal women. Similarly, for the overall psychosocial domain of MenQoL, a significant difference was detected between premenopausal and early perimenopausal women (p=0.004) and postmenopausal and early perimenopausal (p<0.001) women. Early perimenopausal women’s psychosocial domain of quality of life was significantly worse than postmenopausal women and premenopausal women. No significant differences were found in the level of resilience, self-efficacy, and the severity level of how bothered women were by the self-reported experience of poor memory between the menopausal stages (p>0.005).

Using a GLM with age as a covariate showed that menopausal stage was no longer a significant factor in how bothered women were by anxiety and stress between the different menopausal stages. However, the relationship between depression and the total psychosocial domain *vs* menopausal stage remained significant with the inclusion of age as a covariate. Due to the violation of assumptions for parametric analysis for anxiety and depression (but not stress or total psychosocial domain), these analyses need to be regarded with caution.

A partial correlation was run between perceived stress, resilience, self-efficacy, and the severity level of how bothered women were by feelings of depression, anxiety, and memory, whilst controlling for menopausal stage and/or age and is presented in **Table 3**. A statistically significant negative correlation between self-efficacy, resilience, and the severity level of how bothered women were by feelings of anxiety and depression was observed. Only the level of perceived stress was associated with the experience of poor memory.

**Table 3:** Partial correlation between perceived stress, resilience, self-efficacy, severity level of how bothered women were by feelings of depression, anxiety, and poor memory, whilst controlling for age and menopausal stage.

	Perceived Stress	Self-efficacy	Resilience
Perceived stress		-.442	-.438
Depression	.560	-.237	-.262
Anxiety	.483	-.175	-.290
Poor memory	.266	Ns	ns

ns, not significant.

## Discussion

The present study explored the prevalence, and the perceived severity level of how bothered women were by the experience of depression, anxiety, poor memory, *vs*. low perceived stress, resilience, and self-efficacy across the menopausal transition. The results revealed that women in the early perimenopausal phase experienced higher stress levels and were more severely bothered by feelings of anxiety than postmenopausal women. However, when age was taken into account, menopausal stage no longer predicted perceived anxiety or stress levels. This is consistent with the findings of the Study of Women’s health Across the Nation (SWAN)’s longitudinal study in the USA (39). However, Hedgeman et al. (39) found that besides age reducing perceived stress, women with lower educational backgrounds and income also reported a higher level of perceived stress. This association with socioeconomic status variable and perceived stress was not shown in the present UK study.

There are multiple speculative rationales for the observed decline in perceived stress with age. Studies show that middle-aged and older adults possess more coping resources than younger individuals, with increasing life satisfaction, which is inversely correlated with perceived stress (45–47). Furthermore, older adults demonstrated advanced emotional regulation skills (48), which leads to a greater sense of optimism and fewer symptoms of psychological distress than those seen in younger adults (49, 50). However, most studies to date are cross-sectional, which cannot eliminate the possibility of a cohort effect based on the time of birth and associated life experiences, differences in upbringing and societal values related to expression of emotional distress.

A meta-analysis by de Krujf et al, of 11 studies suggested that perimenopausal women (using STRAW criteria) were at not a higher risk of experiencing a clinical depression compared to premenopausal women, but did report more severe symptoms of depression using different scales such as the Center for Epidemiologic Studies for Depression scale (CES-D), Diagnostic and Statistic Manual of Mental Disorders (DSM), Hospital Anxiety and Depression Scale (HADS), Zung self rating depression scale (Zung), and Hamilton Depression Rating Scale (HDRS). However, heterogeneity was not significantly related to type of questionnaire used (30). In this meta-analysis, however, perimenopausal women did not differ from postmenopausal women. Our study found that early perimenopausal women were more severely bothered by feelings of depression, supporting the notion of peri (but not post-) menopausal depression. This result aligns with the concept that there is a specific time frame, known as the “window of vulnerability,” where middle-aged women are perhaps more prone to developing depression (51)—further suggesting that this ‘window’ may refer to the early stages of perimenopause. However, unlike de Kruif et al. (30) meta-analysis, the present study’s data indicated a significant difference in the severity level of how bothered women were by feelings of depression between peri- and postmenopausal women as well, suggesting that these symptoms may reduce over time, after the menopause. The scales and questionnaires used in the studies of the meta-analyses are tailored to help identify depressive symptoms, whereas the MenQoL (while validated) was designed to measure how affected women were by all menopausal symptoms and is not restricted to only psychological complaints, symptoms and identification of pathological disorders using cut-off scores. For instance, sleep issues are included but not as specific to depressive symptoms in the MenQoL.

Mixed results were also found across other cross-sectional and longitudinal studies, as shown in **Table 4**. In the longitudinal studies, women in the (late) perimenopausal stage had the highest odds of experiencing symptoms of depression (18, 53). For instance, Bromberger et al.’s (18) longitudinal study was not included in the meta-analyses (30) but had used the CES-D depression scale. Higher scores on this scale were seen in late peri- compared to early perimenopause, which contrasted with the present study which did not find a significant difference between early peri- and late perimenopausal women in perceived severity of bother by feelings of depression. However, we had low numbers of participants in the late perimenopausal stage which could be related to not finding the same results. Cross-sectional studies generally reported mixed results in differences in symptoms of depression across menopausal stages (with 2 studies finding no difference by stage, one finding higher depression in peri compared to post, and one study finding highest rates in the postmenopausal stage). These studies compared different cohorts of women (25, 26, 52, 54) and differences in life histories and/or demographics in these cohorts could again be partly responsible for these findings.

**Table 4:** Characteristics of recent articles with the assessment of depression or depressive symptoms and stage of the menopausal transition.

Source	Country	Study Design	Instrument for menopausal stage	Instrument for depression	Findings
Almeida et al., 2016 (25)	USA	Cross-sectional	Bleeding pattern	PHQ-9, HADS	No difference between menopausal stage
Zang et al., 2016 (26)	China	Cross-sectional	Bleeding pattern	SDS	No difference between menopausal stage
Pimenta et al., 2016 (52)	Portugal	Cross-sectional	STRAW criteria	DASS	High prevalence of depressive symptoms in peri- compared to post-menopause
Campbell et al., 2017 (53)	Australia	Longitudinal	STRAW criteria	CESD-Brief, Affectometer 2	Peak in the prevalence of depressive symptoms during the perimenopause
An et al., 2022 (54)	Korea	Cross-sectional	STRAW criteria	CES-D	Prevalence of depressive symptoms advances with menopausal stage

PHQ-9, Patient Health Questionnaire; HADS, Hospital Anxiety and Depression Scale; SDS, Self-Rating Depression Scale; CES-D, Center for Epidemiological Studies-Depression Scale; DASS, Depression Anxiety Stress Scale.

The STRAW criteria, which were used to classify menopausal transition stages, were used in other recent studies finding similar effects (30, 52, 53). Mixed findings are thus mainly hypothesised be ascribed to the heterogeneity of the assessment of depression across studies and perhaps comparing different cohorts in cross-sectional studies (23). The present study measured how bothered women were with feelings of depression using MenQoL, instead of using diagnostic criteria indicating a clinical diagnosis of depression to represent a larger range of symptoms experienced rather than using a cut-off of clinically depressed *vs* not depressed. The MenQoL in previous studies was found to be a valid measurement to help identify menopausal women with psychological complaints such as depression and anxiety (55). However, this scale does not allow identification of clinical depression, which (as said) should impact significantly on activities of daily life. The fact that using MenQol, women in early perimenopause reported on average a 4.8 in perceived feelings of depression (on a 1 ‘not at all’ to 8 ‘extremely bothered’, compared to around 3 premenopausally and 3.6 postmenopausally), suggested that on average, women were substantially bothered. The score of 2 would indicate that they experienced feeling depressed, but were not bothered by it, while a score around 3 indicated women were a little bothered (which was the case for women in pre- and postmenopausal stages).

Our data suggested that in women who were post/past the menopause, the experience of depression was at a similar level as to those in the premenopausal stage. However, in South Korean and Italian women, a higher prevalence of depression was seen in postmenopausal women compared to premenopausal women. This discrepancy may be due to the difference in cultural-ethnic (the catholic emphasis on women’s role and child bearing in Italy, for instance, and/or perhaps consumption of different foods in both) and other demographics (education, weight etc) and/or types of (translated) assessment used, as the majority of the women in the present study were from the United Kingdom (81.2%), and most of them were white.

Apart from cultural/racial differences, the number and type of life events and stressors during the menopausal transition are closely related to one’s psychological wellbeing and need to be taken into account in future work (11, 24, 35). The relationship between age, (more severe or clinical levels of) depression using more extensive questionnaires, and menopausal stage also needs to be further explored, as Campbell et al. (53) found that an increasing age had a more substantial influence on reducing depressive symptoms using the CES-D than the reproductive stage itself. Unlike Campbell et al. (53), in our study, menopausal stage remained a significant predictor of perceived severity of bother by feelings of depression after accounting for age. As said, differences in assessments may be responsible for discrepancies. Reviews by Hickey et al. (31) and Hogervorst et al. (56) suggested that there was no increased risk for clinical depression after menopause, except perhaps for women who had a prior history of depression when this is assessed binary (so depression present ‘yes’ or ‘no’).

The effectiveness of treatments for severe psychological complaints during the menopausal transition, such as cognitive behavioural therapy (CBT) and hormone replacement treatment (HRT) with oestrogens, requires further research as the long-term benefits of these treatments are uncertain (56). Further data analysis between vasomotor symptoms, physical symptoms including sleep, pain and depression are required to explore the idea of perimenopausal depression as a unique temporary subtype of depression (21, 23, 57).

Age explained the association of both stress and anxiety in the perimenopausal stage in our study. This data contrast those of the longitudinal study by Bromberger et al. (58), where a higher odds of heightened anxiety levels were seen in (late) perimenopause compared to post-menopause. The discrepancies may be attributed to the lack of standardised measures for anxiety in the present study. *Post hoc* power analyses suggested that our sample size was not an issue (alpha>90%). Whether women in menopausal transition may have a higher risk of developing subsequent clinical mental health disorders, included severe depressive and anxiety disorders using continuous rather than binary data, needs further exploration in a larger sample (31, 57). Our data suggested that improved resilience and self-efficacy and lowering stress could be used to address psychological issues in women across different stages of menopausal transition and are independent of age. Whether CBT mediates improved mood through its effect on resilience, self-efficacy or stress reduction needs to be investigated in perimenopausal women.

Perceived stress was the only factor associated with perceived memory issues. However, how bothered women were by the experience of poor memory was not different between menopausal stages. It has been suggested that a subsample of women (around 23%) does experience objective memory impairment, but this is certainly not the case for the majority of women (56). Woods et al. (59) found that US-based menopausal women’s daily perceived stress was significantly more influenced by being employed, having a depressed mood, and perceiving one’s health as poor, rather than the severity of hot flashes (named flushes in the UK). In addition, in their study, social support was significantly lower during the early perimenopause stage than in other stages, allowing a potential intervention at that particular stage. How these impacts on perceived and objective cognitive performance needs to be investigated and whether CBT could benefit this.

Cognitive Behavioural Therapy or CBT, which includes coping strategies for women to learn how to cope with symptoms throughout the transition, was the most effective in reducing negative moods in a group settings for menopausal women (60). Quoting de Kruif et al. (30), *“the menopausal transition may be seen as the straw that breaks the camel’s back.”* The spike in level of how bothered women were by feelings of depression experienced in early perimenopause related to changes in hormone levels may be attributed to increased stress (further reducing memory performance) and anxiety levels with the onset of menopause and the psychosocial issues experienced at that age, such as increased pressures at work, family changes (care for older parents, death, teenagers, empty nest syndrome, possible marital issues related to this stress and reduced sexual libido etc).

Future studies should consider appraisal of the menopausal transition, adverse life experiences, and social support when investigating psychological complaints and stress in menopause. Moreover, future intervention studies targeting psychological complaints should incorporate self-efficacy and resilience, as other studies have shown that psychological resilience may mediate the relationship between general self-efficacy and mental health (61).

### Limitations

There are some limitations in this study that need to be addressed. First, due to the study’s cross-sectional design, the causal relationship between stress, depression, anxiety, and menopausal stages could not be determined. Data on the psychiatric history of participants, which is known to be one of the risk factors for the occurrence of depression during the menopause transition, and potential use of antidepressants, anxiolytic drugs, and phytomedicine, which may act as a confounding factor were not collected. Due to the small sample size, we did not find any significant differences between surgical and natural menopause women and perhaps between early and late perimenopausal stages, which is inconsistent with existing literature (56). Finally, the data was collected through an online survey relying on self-report, which may be subject to biases and misinterpretation of questions. Using better questionnaires for continuous scaling of all depressive and anxiety symptoms would be suggested for future studies including past and medical and psychiatric history, following the same women over time.

## Conclusion

Middle-aged women undergoing menopausal transition in this study were most stressed, depressed, and anxious in the early perimenopausal phase. Rather than the menopausal stage. however, differences in age between women explained the severity level of how bothered women were by feelings of anxiety and stress levels, whereas this was not the case for depression. The relationship between perceived stress, protective factors such as self-efficacy, and resilience, and their impact on mood during the menopausal transition needs further exploration alongside factors such as social support and life events. Mid-life is a critical time to provide support and help women to enhance their quality of life, especially as the early perimenopausal stage is often not recognized by women. Further research is needed to understand this relationship better and to develop more effective interventions to help women manage their mental health during the menopausal transition.

## Data availability statement

The raw data supporting the conclusions of this article will be made available by the authors, without undue reservation.

## Ethics statement

The studies involving humans were approved by Loughborough University Ethics Review Sub-Committee. The studies were conducted in accordance with the local legislation and institutional requirements. The participants provided their written informed consent to participate in this study.

## Author contributions

MK: Conceptualization, Data curation, Formal analysis, Investigation, Methodology, Visualization, Writing – original draft, Software. EH: Supervision, Writing – review & editing, Formal analysis.
