# Supplementary material for: Stress, depression, and anxiety: psychological complaints across menopausal stages
Source: Front Psychiatry. 2024 Feb 22;15:1323743. doi: 10.3389/fpsyt.2024.1323743 (PMC10917984; doi:10.3389/fpsyt.2024.1323743)
Supplement: Supplementary file 1 [file Table_1.docx]

Supplementary Material

Stressed, depressed, and anxious: going through the change and psychological complaints in menopause.

MingJun Kuck*, Eef Hogervorst

*** Correspondence:** MingJun Kuck: m.j.kuck@lboro.ac.uk

**Supplementary Table 1.** *Type of menopause and level of perceived stress, and how bothered women were by feelings of depression, anxiety, and memory issues as measured by the Menopausal Quality of Life Scale.*

|  | Mean *(SD)* | |
| --- | --- | --- |
|  | Natural menopause | Surgical menopause |
| - Age | 51.57*(4.61)* | 53.85*(4.23)* |
| - Education | 2.84*(0.84)* | 2.95*(1.05)* |
| - Perceived Stress | 20.85*(7.41)* | 19.84*(6.70)* |
| - Feelings of depression | 4.08*(2.62)* | 4.20*(2.31)* |
| - Feelings of anxiety | 4.64*(2.60)* | 4.63*(2.80)* |
| - Poor memory | 4.52*(2.52)* | 4.75*(2.36)* |
